# Hearing Loss due to Infiltration of the Tympanic Membrane by Chronic Lymphocytic Leukemia

**DOI:** 10.1155/2012/589718

**Published:** 2012-11-07

**Authors:** Jonathon B. Cohen, Robert Cavaliere, John C. Byrd, Leslie A. Andritsos

**Affiliations:** ^1^Division of Hematology, The Arthur G. James Comprehensive Cancer Center and The Ohio State University, 320 W 10th Avenue, B354 Starling Loving Hall, Columbus, OH 43210, USA; ^2^Department of Neurosurgery, The Arthur G. James Comprehensive Cancer Center and The Ohio State University, Columbus, OH 43210, USA

## Abstract

Central nervous system (CNS) involvement by chronic lymphocytic leukemia (CLL) can present with dramatic neurologic findings or can be quite subtle, discovered only at the time of autopsy. We describe a case of CLL in a patient who presented initially with hearing loss and was ultimately found to have involvement of the tympanic membrane. She noted improvement of her hearing after induction therapy but was not aware at the time of the involvement of her CNS with CLL. Upon worsening of hearing at the time of relapse, she was evaluated by imaging and CSF analysis as well as biopsy of the tympanic membrane, and involvement of the CNS was confirmed. She has received CNS-directed therapy with intrathecal liposomal cytarabine and intravenous CNS-directed therapy and has noted improved hearing and resolution of her imaging and CSF findings. This is the first reported case of tympanic membrane involvement with CLL and describes potentially effective methods for managing this challenging complication.

## 1. Introduction

Central nervous system (CNS) involvement by chronic lymphocytic leukemia (CLL) is a rare complication, with an incidence of 8% found at autopsy but with far fewer patients diagnosed while alive [1]. Patients can present with headaches, mental status changes, cranial nerve abnormalities, optic neuropathy, lower extremity weakness, or cerebellar signs [2]. Others will have more subtle findings which may be difficult to identify. The prognosis of patients with CNS involvement is thought to be poor compared to nonaffected counterparts, although data remain limited to case reports. A recently published compilation of previously reported cases found a median overall survival from the time of documented CNS involvement of about 12 months. Patients can develop CNS involvement at any Rai stage and published reports indicate involvement of the CNS at any time during the course of the disease, from initial presentation to 14 years after initial diagnosis [3]. In this report, we describe a case of CLL involving the tympanic membrane that presented with hearing loss. To our knowledge, this is the first reported case of CLL involvement of the tympanic membrane.

## 2. Case Presentation

A 66-year-old female with no significant past medical history presented to her primary care physician for evaluation of progressive bilateral hearing loss. This was initially attributed to bilateral otitis media and sinusitis, and she was referred to an otolaryngologist for evaluation. Myringotomy tubes were placed without improvement in her symptoms. On re-evaluation, a tympanic membrane biopsy was performed which revealed involvement by a monotonous population of B-lymphocytes consistent with SLL/CLL. Immunohistochemical staining demonstrated a population of cells positive for CD20, CD5, and CD23 that were negative for CD10 and Cyclin D1. A complete blood count (CBC) at that time demonstrated a white blood cell count of 104,000/*μ*L. Peripheral blood immunophenotyping was consistent with a diagnosis of chronic lymphocytic leukemia. Peripheral blood cytogenetic analysis was not performed. She initially was managed expectantly; however she developed worsening lymphocytosis and lymphadenopathy. Several months later she was treated with rituximab, cyclophosphamide, vincristine, and prednisone (R-CVP). She completed 6 cycles of therapy with a partial response but noted marked improvement in her hearing. She experienced progression of her disease 3 months after stopping therapy and was treated with 4 doses of weekly rituximab which resulted in stable disease. At that time, she was referred to our institution for further management.

**Figure 1:**
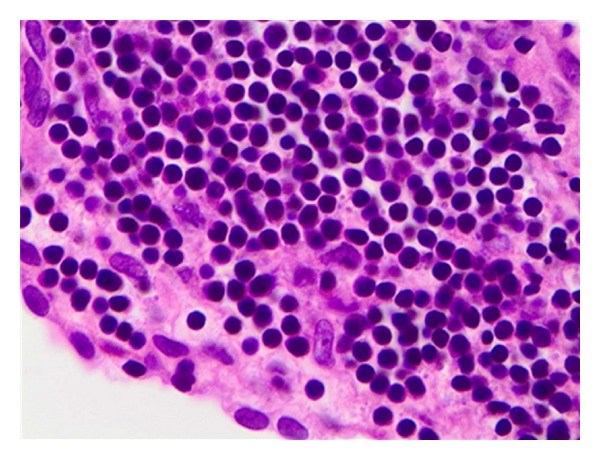
Hematoxylin and eosin staining of the tympanic membrane, demonstrating infiltration with mature-appearing lymphocytes, consistent with involvement with CLL.

**Figure 2:**
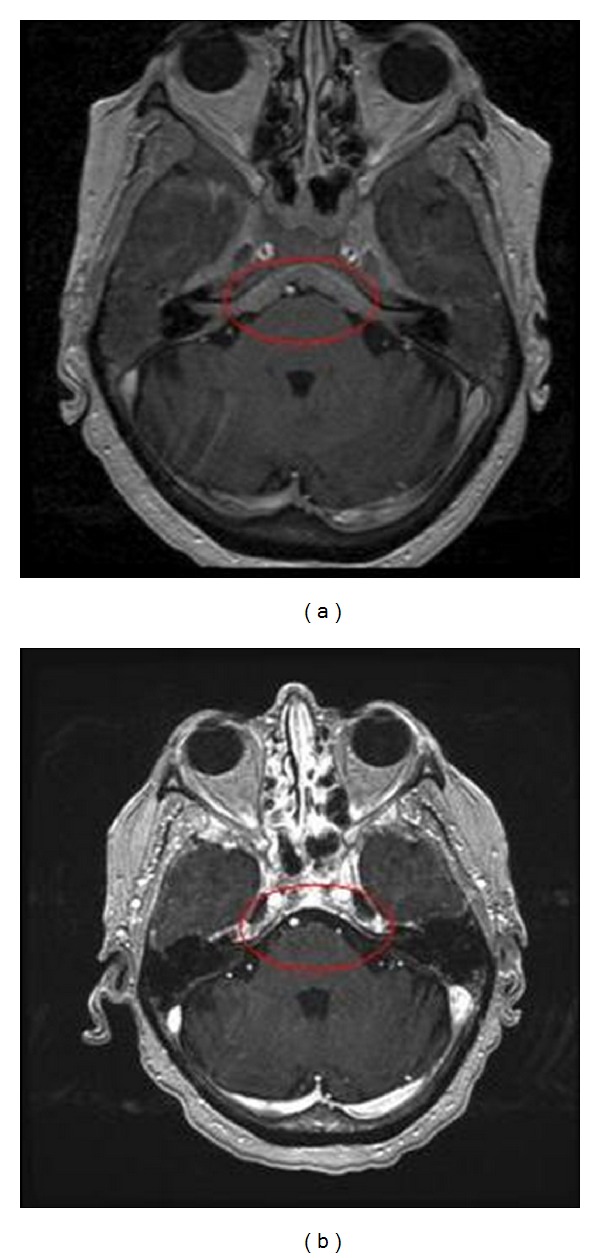
Magnetic resonance imaging of the brain demonstrating thickening of the skull base (a) that has responded to therapy and appears normal in subsequent imaging (b).

At the time of her initial visit, she was experiencing fatigue and worsening of her hearing loss. CBC showed a white blood count of 115,600/*μ*L, a hemoglobin of 10.5 g/dL, and a platelet count of 348,000/*μ*L. A review of a prior bone marrow biopsy confirmed the diagnosis of CLL and her peripheral blood cytogenetic profile demonstrated del(17p13.1) and del(13q34). The tympanic membrane biopsy was reviewed and involvement by CLL was confirmed (see Figure 1). A brain MRI showed bony and soft tissue enhancement along the skull base including the mandible, maxilla, and part of the calvarium with intracranial extension (see Figure 2(a)). Cerebrospinal fluid (CSF) evaluation confirmed the presence of leptomeningeal disease. CT scans revealed generalized adenopathy including the bilateral axillae, mediastinum, celiac, retroperitoneal, and iliac regions, without hepatomegaly or splenomegaly. 

As she wished to defer chemotherapy, she was initiated on therapy with methyprednisolone (1 gram/m^2^ daily for 3 days) and rituximab (375 mg/m^2^ weekly for 12 weeks) as described by Castro et al. [4]. In addition, she received intrathecal liposomal cytarabine, but her course was complicated by arachnoiditis requiring the intrathecal therapy to be held after three doses. At the conclusion of 12 weeks of systemic therapy, she achieved a partial response along with clearing of her CSF. She had an initial improvement of her symptoms, but within 3 months developed recurrent hearing loss associated with dizziness and imbalance. At this time, she was treated with cyclophosphamide, cladribine, and rituximab (CCR), given the central nervous system penetration of cladribine, as well as concurrent intrathecal liposomal cytarabine [5, 6]. She achieved only stable disease and remained symptomatic. She next was treated with HyperCVAD, and at the conclusion of 4 cycles she demonstrated marked improvement in her nodal disease and peripheral blood lymphocytosis. In addition, her CNS MRI abnormalities significantly improved (see Figure 2(b)), as did her hearing loss and dizziness. She was evaluated for a reduced-intensity conditioning allogeneic transplant and her sister was found to be HLA identical. However, after consideration of options, she chose not to proceed with transplantation. She has remained off of treatment for 12 months without overt evidence of progression and without recurrence of her hearing loss. 

## 3. Discussion

 While CLL involvement of the CNS is generally associated with advanced disease, one recent report describes a patient with brain involvement as the only site of disease. This patient had concerning MRI findings and biopsy confirmed infiltration with clonal B-lymphocytes consistent with CLL/SLL [7]. In the absence of lesions which are accessible for biopsy, the most common way to evaluate for leptomeningeal disease is by CSF analysis. This can be challenging as malignant lymphocytes are often indistinguishable morphologically from nonmalignant lymphocytes that may be present in the CSF for other reasons (i.e., inflammation). Therefore, assessment of CSF with both cytologic and immunophenotypic assessments is important to document the presence of malignant lymphocytes [8]. In the presented case, cytologic evaluation yielded a diagnosis of “lymphocytosis” while immunophenotypic assessment confirmed the involvement of CLL. Imaging studies such as CT or MRI can also indicate involvement, although there have been several cases reported of documented leptomeningeal involvement without imaging abnormalities [3].

There is no defined therapy for patients with CNS involvement. In one published series of 5 patients, all patients received intrathecal therapy. Four patients were treated with intrathecal methotrexate and cytarabine, while the other was treated with methotrexate alone. In addition, three out of the five patients received whole brain radiation therapy (WBRT). Three patients received systemic therapy comprised of chlorambucil or CHOP. None of the 5 patients achieved complete resolution of their CNS involvement, although four patients experienced prolonged survival [9]. Calvo-Villas and others report two patients with CLL who were treated with intrathecal liposomal cytarabine in conjunction with systemic therapy and achieved complete resolution of neurologic symptoms and remission of CNS disease. Neither patient experienced a CNS relapse [10]. In a compilation of published reports, 69% (9/13) of patients treated with radiation therapy alone achieved a durable remission, compared to 76% of patients treated with intrathecal chemotherapy alone, and 85.7% of patients treated with intrathecal chemotherapy and radiation [3]. Management of CNS involvement by CLL continues to be a challenge, and there are limited data to guide therapy. Intrathecal chemotherapy may effectively clear leptomeningeal disease, and systemic chemotherapy with CNS penetration may augment this approach. Large, discrete lesions may require the addition of radiation therapy. Our patient achieved excellent disease control with systemic chemotherapy with HyperCVAD and intrathecal liposomal cytarabine. Studies of this unusual disease complication are warranted but would likely require a cooperative effort by multiple institutions given the rarity of CNS involvement by CLL.
